# Factors influencing work participation of adults with developmental dyslexia: a systematic review

**DOI:** 10.1186/1471-2458-14-77

**Published:** 2014-01-24

**Authors:** Joost de Beer, Josephine Engels, Yvonne Heerkens, Jac van der Klink

**Affiliations:** 1 Department Occupation & Health, HAN University of Applied Sciences, Nijmegen, The Netherlands; 2 Department of Health Sciences/Community and Occupational Medicine, University Medical Center Groningen, Groningen, The Netherlands

**Keywords:** Adult, ICF, Developmental dyslexia, Work participation

## Abstract

**Background:**

Evidence has been synthesized to determine hindering and facilitating factors associated with the work participation of adults with developmental dyslexia (DD), classified according to the International Classification of Functioning, Disability and Health (ICF).

**Methods:**

A systematic literature review has been performed. Two search strings were used to determine the population and the context of work. The ICF was expanded with two subdivisions: one that made the environmental factors more work-related and a subdivision of personal factors. For data extraction the method known as qualitative metasummary was used and the manifest frequency effect size (MFES) for each category in the ICF was calculated.

**Results:**

From 33 included studies 318 factors have been extracted and classified in the ICF. In the classification the frequency of occurrences and the consistency in direction (i.e., hindering or facilitating) have been made visible. The ICF categories with the highest MFES were *mental functions* with factors like feelings and emotions about dyslexia; *activities* like reading or writing/spelling; *participation* with factors like acquiring and keeping a job; *social relationships at work* where the attitudes and support of the employer and co-workers are important; *working conditions* with factors like the availability of assistive technology and accommodations on the job; and *personal factors* like self-disclosure and coping strategies.

**Conclusions:**

In the context of work DD affects nearly all domains of functioning, mostly in a negative way. Within each domain the impact of DD increases over the course of life. To overcome that negative influence, many forms of adaptation, compensation, or coping are mentioned. Also notable is the lack of positive attitudes toward DD of the participants with DD—with the exception of the attitudes of teachers with DD—as well as on the part of colleagues, supervisors, and employers.

## Background

Developmental dyslexia (DD) is a disorder that affects reading and spelling development. DD is usually associated with impairments in phonological processing, verbal processing speed and verbal short-term memory [1,2]. It makes reading and spelling difficult for children who otherwise possess the intelligence and motivation considered necessary for accurate and fluent reading [3]. Children with DD develop weak literacy skills; hence, its great impact on education. In the course of children's development the range of difficulties becomes wider: besides problems in cognitive functioning, like trouble with reading, spelling, memory, and word finding, some executive difficulties arise, such as clumsiness, problems in organizing activities, and poor time management [4]. Experiencing these difficulties will affect the self-image and self-awareness of children and adolescents with DD, although they could also become persevering and goal-driven [5]. Whatever their reaction, young people with DD develop a variety of coping strategies and adaptation mechanisms that may account for individual differences as adults [4].

It is known that 80% of the children and adolescents identified as having learning disabilities have DD [3]. In both a Dutch and an American population-based study the prevalence of DD among children at the end of their primary education is about 4–5% [6,7]. These percentages reflect the prevalence rates from 2–11% mentioned in the International Book of Dyslexia [8], in which a short description of dyslexia in 54 countries is given. The figures mentioned above are indicative because it is very difficult to find exact prevalence figures about adults with DD and because these figures are strongly related to the definition of learning disabilities/developmental dyslexia that has been chosen, and from which the criteria used to construct the prevalence rates are extracted.

Despite difficulties, most young adults with DD enter the labor market after completing their school careers. In the past two decades the educational possibilities for children and adolescents with DD have been improved through assistive technology, by giving them extra time, and other adaptations, like recorded textbooks or a bigger font [5]. At work, however, these improvements are not so obvious as in education. More and more generally well-educated young people who have learned to cope with DD within the context of support and adaptations enter a workplace that is not as well prepared for employees with DD as educational institutions are. When people with DD are employed, they often have to re-adapt to the difficulties they had struggled with and overcome in education [9]. On top of persistent difficulties in reading, writing, short-term-memory, and processing speed [9], they encounter new problems specific to the context of work. According to the Van Dijk model of workload and capacity [10], relevant factors in the work context can be subdivided into four categories: work content, work circumstances, terms of employment, and relationships at work. Some examples of problems for employees with DD are complex tasks that cause much workload (work content) [11] and the dependence on social support from colleagues (relationships at work) and/or from family members at home [12]. Task content and relations with and attitudes of colleagues and employer can intensify or temper the nature of the problems [13]. The physical work environment, e.g., being subjected to noise or other distractions, may also cause difficulties [11].

Besides its possible negative effects, there may also be a positive side to DD. The highly visual way of thinking that is characteristic of many people with DD, is helpful in problem solving [12]. Employees with DD tend to recognize patterns of information quickly and can mentally rearrange designs and processes [14]. Moreover, many people with DD have great perseverance in combination with high ambition and strong motivation [15].

The positive and negative characteristics of employees with DD on the one hand, and the variety of coping strategies and adaptation mechanisms on the other hand, result in different career patterns. Examples abound of adults with DD who have become great entrepreneurs [16]. However, there are also many instances of misfortune and failure in the world of work [17]. This duality raises the question, what explains these differences in patterns of gainful employment? Before attempting to answer this question, the factors associated with work participation of adults with DD should be more fully understood. To that end, a systematic literature review has been performed, resulting in the identification of positive and negative factors related to DD and employment. The International Classification of Functioning, Disability and Health (ICF) [18] was used to classify those factors. The ICF offers a framework in which functioning and health is a result of a complex interaction between and among body functions and body structures, activities and participation, and personal and environmental factors. The ICF has a broad scope both at the individual as well as the societal level. It offers a comprehensive list of categories necessary for the description of functioning [19].

### Aim of the systematic review

The aim of this systematic review was to determine hindering and facilitating factors associated with participation in work of individuals with DD, classified according to the dimensions of the ICF.

## Methods

As in the US-literature learning disabilities is the umbrella term that includes developmental dyslexia as well, the definition of learning disabilities was chosen as starting point for the literature search. For the aim of this review the definition of learning disabilities developed by the National Joint Committee on Learning Disabilities seems most fitting [20] (p.20): *“Learning disabilities* is a general term that refers to a heterogeneous group of disorders manifested by significant difficulties in the acquisition and use of listening, speaking, reading, writing, reasoning or mathematical abilities. These disorders are intrinsic to the individual, presumed to be due to central nervous system dysfunction, and may occur across the life span. Problems in self-regulatory behaviors, social perception, and social interaction may exist with learning disabilities but do not by themselves constitute a learning disability. Although learning disabilities may occur concomitantly with other handicapping conditions (for example, sensory impairment, mental retardation, serious emotional disturbance), or with extrinsic influences (such as cultural differences, insufficient or inappropriate instruction), they are not the result of those conditions or influences.” An additional benefit of this definition is that it contains several dimensions that correspond to the ICF, used as a framework in this study: the disorder is “presumed to be due to central nervous dysfunction” (functions and structures). Furthermore it refers to “difficulties in the acquisition and use of listening, speaking, reading, writing, reasoning or mathematical abilities” (activities). The disorder may cause “problems in self-regulatory behaviors, social perception and social interaction” (participation). Finally the disorder may “occur with other handicapping conditions or extrinsic influences” (environmental factors). Also the time dimension—“these disorders may occur across the life span”—fits with the aim of this review.

As indicated the definition of learning disabilities in the U.S. literature includes developmental dyslexia as well. Both terms—learning disabilities and developmental dyslexia—are incorporated in the search strings. To limit the review to dyslexia we included only those studies that had the word ‘dyslexia’ in the Methods or the Results section.

### Identification of studies

The relevant literature was identified by performing searches in the electronic bibliographical databases Medline, Academic Search Premier, Cinahl, PsycInfo, Cochrane Central Register of Controlled Trials, Web of Science, Science Direct, and Embase. The searches were limited to primary studies, published from 1995 forward. This year was chosen as the starting date for two reasons. First, dyslexia was redefined around that time in the United States and in several European countries. Second, legislation was enacted at that time in the United States, Canada and the United Kingdom to protect the rights of people with disabilities at work.

*To determine the population,* we searched for the following MeSH terms (Medical Subject Headings) and free text words in the title and abstract: (dyslexia[MeSH] OR alexia*[MeSH] OR "word blindness" OR “developmental reading disorder*”[MeSH] OR “developmental reading disabilit*”[MeSH] OR “learning disorder*”[MeSH] OR “learning disabilit*”[MeSH]) AND (word* OR read* OR writ* OR language*).

*To determine the context of work*, we searched for the following MeSH terms and free text words in the title and abstract: (employment[MeSH] OR occupation*[MeSH] OR work[MeSH] OR job*[MeSH] OR participat* OR "task performance and analysis"[MeSH] OR "job satisfaction"[MeSH] OR "career choice"[MeSH] OR "work status" OR "employment status" OR absenteeism[MeSH] OR "sick leave"[MeSH] OR “disability evaluation"[MeSH]) AND (work* OR job* OR occupation* OR employ* OR "return to work" OR "rehabilitation, vocational"[MeSH]).

These search strings were used in Medline. They were modified for use in the other databases, when different search terms were used. In all databases the searches were done with the strings for ‘dyslexia’ AND ‘work’.

The set that was used to include studies consisted of four criteria:

A. *Population*

1. ‘Dyslexia’ or ‘(specific) learning/reading disorder/disability’ mentioned explicitly in the title or abstract.

2. Addressing working population, aged 18 to 65 years.

B. *Method*

3. Primary research paper of quantitative or qualitative methodology, published after 1995, in English, German or Dutch.

C. *Outcome*

4. Focusing on the relation between dyslexia or (specific) learning/reading disorder/disability and work.

Studies were included if they met all four criteria. The criteria were not weighted.

### Review procedure

Titles and abstracts of the studies identified through the search strategy were screened independently by two authors (JdB and JE). Studies that did not meet the inclusion criteria were discarded. When the two authors disagreed, even after a consensus meeting, a third reviewer (YH) was consulted.

A study was selected for full-text examination if in the title or abstract:

•it was unclear whether it was a primary study;

•it was unclear whether adults with dyslexia were meant by the descriptor ‘people with learning disabilities’ or ‘(learning) disabled people’. The word ‘dyslexia’ had to be present in the Methods or Results section;

•it was unclear if the population was still studying or already had a job;

•reference was made to an activity, a personal factor, an environmental factor or a mental function, but without an explicit link to work.

Two reviewers (JdB and JE) independently scanned the full texts of these studies to determine if there was additional information available to clear up the uncertainties mentioned above. If any disagreement remained after a consensus meeting, a third reviewer (YH) was consulted who made a definitive choice to include or exclude the study.

Once the set of eligible studies had been compiled, the reference lists of all studies were searched for additional studies (the snowball method). The indexes of all volumes published in the period covered by this review of the journals in which the included studies were published were also searched.

### Quality assessment

Before the quality assessment a distinction was made between qualitative and quantitative studies. This was done because of the nature of the factors expected to be found in both categories of the studies: The subjective factors in the qualitative studies are hard to measure quantitatively, and the quantitative studies have more quantifiable factors. Moreover, there are different instruments to assess the quality of both categories.

To assess the quality of qualitative studies two reviewers (JdB and JE) independently used the seven criteria from the handbook of the Netherlands Quality Institute for Health Care, CBO [21]. These criteria are based on questions listed by Mays and Pope for assessing the quality of qualitative studies [22]. Each study was graded on the criteria from this list as ‘+ = present’, ‘- = not present’ and ‘± = insufficiently described’, without passing a final judgment on its quality. A minimum level of quality as defined by the CBO [21] is at least five ‘+’, and one ‘±’. If a study did not meet that criterion, it was still used to extract factors as an additional ‘check’ (see the Results section).

For quantitative studies the two reviewers used the STROBE Statement, a checklist of 22 items that any report of quantitative cross-sectional studies should contain [23]. This particular checklist was chosen because we did not expect to find other kinds of publications with a quantitative methodology, such as randomized clinical trials (RCT’s) or cohort studies, among the selected works. Although it is a report checklist, it was used to assess the quality of the studies because reporting and quality assessment in our view are two sides of the same coin. As for the qualitative studies all criteria on the list were graded as ‘+ = present’, ‘- = not present’ or ‘± = insufficiently described’. The minimum level of quality was chosen in analogy with the criterion used in the qualitative study: at least 15 ‘+’, and three ‘±’, as the amount of items in the checklist is approximately three times higher than the amount of criteria used to determine the quality of the qualitative studies. If a study did not meet that criterion, it was still used to extract factors as an additional ‘check’ (see the Results section).

To measure interrater reliability at the level of the criteria of both the qualitative and quantitative studies, Cohen’s Kappa [24] was calculated.

### Data extraction

All included studies were assessed in terms of factors associated with the work participation of adults with DD. A factor is a single element or a construct that has a positive or negative influence on work participation. These factors had to be mentioned explicitly in the text. The interpretation of these factors by the study participants, in terms of impediment or facilitator, will be indicated. In the Results section two dimensions of each factor will be made visible—the frequency of mentioning and, if a factor is mentioned more than once, the consistency: Is a factor labelled with a consistently positive or negative impact on work participation in the qualitative studies? In quantitative studies, consistency means that if a factor is involved in the statistical analysis, that factor is considered statistically significant or important.

We used the method known as qualitative metasummary [25] (p.152), which consists of the following techniques:

a. extracting findings, separating them from other elements of the research reports;

b. editing the findings to make them accessible;

c. grouping findings in common topical domains;

d. abstracting findings and classifying them; and

e. calculating manifest frequency. For this purpose the manifest frequency effect size (MFES) is presented. This size is calculated by dividing the number of all studies that met the quality criterion and in which a factor was found (each factor within a study was counted just once) by the total number of studies meeting the quality criterion.

### Data classification

**Figure 1:**
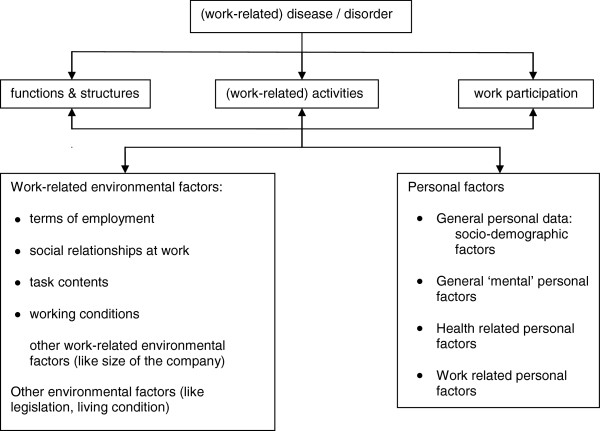
**ICF scheme.** Here the original ICF scheme is expanded with concepts from the model of Van Dijk [26] and from a proposed subdivision of the personal factors [27].

All factors were classified according to the International Classification of Functioning, Disability and Health (ICF) [18]. The expansion of Heerkens et al. [26] was used to close the gap between the terminology used in health fields and in occupational medicine. For that expansion the authors used the model of Van Dijk et al. [10] (Figure 1) to describe the work-related environmental factors. For the personal factors a subdivision was used, proposed by Heerkens et al. [27] at the WHO-FIC annual network meeting in 2012. The category ‘Lifestyle’ in that subdivision was omitted for this review because of its irrelevance. The concepts in Figure 1 are used to classify the factors found in both qualitative and quantitative studies (in the Results section, the qualitative and quantitative studies are presented separately). These factors were linked to the best fitting ICF category. Although the central issue in this review is ‘participation in work’, the factors influencing work participation can be classified in all ICF categories.

The research for this systematic review started in October 2009. The use of the alert function in several databases prevented us from missing relevant studies while working on this review, the most recent included study is of 2013.

## Results

The database searches yielded 2418 studies, 523 of which were duplicates. In total 1895 studies were qualified for screening.

After independent screening of title and abstract, 1801 studies were excluded; 80 studies were selected for full-text scrutiny due to insufficient information in the title and abstract to warrant either inclusion or exclusion; and 14 were deemed eligible (these 14 studies remained eligible after full-text screening). The 80 full-text selections were screened on the four criteria set forth in the Methods section. An independent review by two reviewers on these four criteria resulted in 14 inclusions, 65 exclusions and one study on which the two reviewers disagreed. A third reviewer (YH) was asked to give a final opinion. Because of a lack of clarity about the exact nature of the learning disabilities of the people who had been interviewed, that study was also excluded.

**Figure 2:**
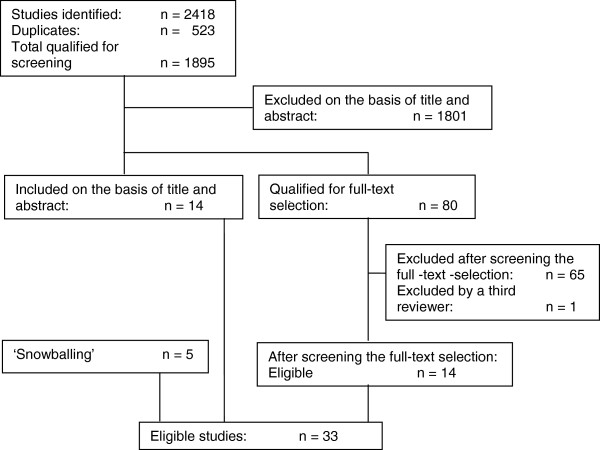
Selection of eligible studies.

The snowballing of the sources cited in the included studies and in past volumes of the journals in which the inclusions had been published yielded another five eligible studies. In total, 33 studies were deemed eligible (See Figure 2). Of these 33 eligible studies, 17 are qualitative and 16 quantitative. These categories will be described separately.

### Methodological quality of the qualitative studies

We used a list from the Dutch Quality Institute for Health Care CBO to assess the methodological quality of the qualitative studies [21]. As indicated each study was graded on the criteria from this list as + = present, − = not present, or ± = insufficiently described. The studies were sorted by number of plusses; those with an equal number of plusses were sorted further by publication year and alphabetical order (see Table 1). A column has been added to Table 1 for the number of factors found in each study.

**Table 1:** Main criteria for assessing the methodological quality of the qualitative studies; the quality of studies with less than 5+ and 1 ± is insufficient

**Author(s)**	**Relevance and goal**	**Method and techniques appropriate**	**Selection of participants**	**Data collection**	**Theoretical framework**	**Data analysis**	**Results**	**Number of factors**	**Number of +/±**
Hellendoorn and Ruijssenaars, 2000 [28]	**+**	**+**	**+**	**+**	**+**	**+**	**+**	73	7+
McNulty, 2003 [29]	**+**	**+**	**+**	**+**	**+**	**+**	**+**	44	7+
Price et al., 2003 [30]	**+**	**+**	**+**	**+**	**+**	**+**	**+**	47	7+
Burns and Bell, 2011 [31]	**+**	**+**	**+**	**+**	**+**	**+**	**+**	72	7+
Ferri et al., 2001 [32]	**+**	**+**	**+**	**+**	**+**	**-**	**+**	20	6+
Lindstrom and Benz, 2002 [33]	**+**	**+**	**±**	**+**	**+**	**+**	**+**	31	6+/1±
Gerber et al., 2004 [34]	**+**	**+**	**±**	**+**	**+**	**+**	**+**	79	6+/1±
Burns et al., 2013 [35]	**+**	**+**	**+**	**+**	**+**	**±**	**+**	55	6+/1±
Raskind et al., 1997 [36]	**+**	**+**	**-**	**+**	**+**	**±**	**+**	116	5+/1±
Shessel and Reiff, 1999 [37]	**+**	**+**	**±**	**+**	**+**	**-**	**+**	43	5+/1±
Price and Gerber, 2001 [38]	**+**	**+**	**+**	**±**	**-**	**+**	**+**	14	5+/1±
Ferri et al., 2005 [39]	**+**	**+**	**-**	**±**	**+**	**+**	**+**	21	5+/1±
Macdonald, 2009 [40]	**+**	**+**	**+**	**±**	**+**	**-**	**+**	33	5+/1±
Duff et al., 2007 [41]	**+**	**+**	**±**	**+**	**-**	**-**	**+**	46	4+/1±
Illingworth, 2005 [42]	**+**	**+**	**±**	**±**	**-**	**±**	**+**	81	3+/3±
Greenbaum et al., 1996 [43]	**+**	**±**	**±**	**±**	**-**	**-**	**+**	25	2+/3±
Gilmour, 1998 [44]	**+**	**-**	**±**	**±**	**-**	**-**	**-**	10	1+/2±

(‘+’ = present; ‘-‘ = not present; ‘±’ = insufficiently described).

As indicated a study should have a minimum of five plusses and of the remaining two items, at most one may be absent and one may be insufficiently described. Of the 17 qualitative studies, 13 reached that threshold.

We measured interrater reliability at the level of the criteria independently scored by the two reviewers. Therefore we calculated a Cohen’s Kappa. For the qualitative studies the Kappa was 0.82.

### Main characteristics of the qualitative studies

Additional file 1 displays the main characteristics of the qualitative studies. For each one, the theoretical framework is specified, the aim of the study is summarized, the method of data collection is indicated, and the participants and the sample size are described.

In three studies the theoretical framework was not mentioned [38,43,44] and in two studies [41,42] the framework was called qualitative or exploratory, but was not otherwise specified. The remaining 12 studies used one of the following frameworks: multiple case study, narrative analysis, ethnographic perspective, discourse analysis, or biographical approach.

The number of participants ranged from 3 to 27, with two outliers of 49 [34,43]. The total number of participants was 256 with an average of 15. Two studies did not give the distribution of gender; in the remaining 15 studies, 134 men (56%) and 104 women (44%) were participants, with a mean age of 33 years. The educational and employment status diverged strongly: 22 were unemployed; 224 were employed in various occupations; and in one study [44] the employment status was not mentioned. The method of data collection in 16 of the 17 studies was an individual, in-depth interview.

In four studies, the focus was on experiencing dyslexia in life in general, part of which is employment [28,29,37,40]. In eight studies, attention was paid to dyslexia in the work situation: two in general [30,43]; five in a specific occupation like teaching [31,32,35,39] or nursing [42]; and one in a specific group, young women entering the workforce [33]. In two studies the position of dyslexic employees was examined after the introduction of protective legislation in the United States and Canada [34,38]. The focus of two studies was on the effects of the introduction of assistive technology on learning disabilities (LD) [36] and of the Anger Management Programme on LD [44]. Only one study examined the employer’s attitude toward LD [41].

### Methodological quality of the quantitative studies

The STROBE Statement was used to assess the methodological quality of the quantitative studies [23]. As indicated for each study all criteria on the list were graded as + = present, − = not present, or ± = insufficiently described. The studies were sorted by number of plusses and ‘±’, those with an equal number of plusses were sorted by publication year and alphabetical order (see Table 2).

**Table 2:** Criteria for assessing the methodological quality of the quantitative studies; the quality of studies with less than 15+ and 3 ± is insufficient

**Author(s)**	**1**	**2**	**3**	**4**	**5**	**6**	**7**	**8**	**9**	**10**	**11**	**12**	**13**	**14**	**15**	**16**	**17**	**18**	**19**	**20**	**21**	**22**	**+/±**	**n fact**
Mellard et al., 2007 [45]	**+**	**+**	**+**	**-**	**+**	**+**	**+**	**+**	**+**	**+**	**+**	**+**	**+**	**+**	**+**	**+**	**+**	**+**	**+**	**+**	**+**	**+**	21+	6
Madaus et al., 2003 [46]	**+**	**+**	**+**	**±**	**+**	**+**	**+**	**+**	**+**	**+**	**+**	**+**	**+**	**+**	**+**	**+**	**-**	**+**	**+**	**+**	**+**	**-**	19+/1±	21
Dickinson & Verbeek, 2002 [47]	**+**	**+**	**+**	**-**	**-**	**+**	**+**	**+**	**+**	**+**	**+**	**+**	**+**	**+**	**+**	**+**	**+**	**+**	**±**	**+**	**+**	**+**	19+ / 1±	15
Rojewski, 1999 [48]	**+**	**+**	**+**	**-**	**+**	**+**	**+**	**+**	**+**	**+**	**+**	**+**	**+**	**+**	**+**	**+**	**-**	**+**	**-**	**+**	**+**	**-**	18+	10
Madaus et al., 2008 [49]	**+**	**+**	**+**		**+**	**+**	**+**	**+**	**-**	**+**	**+**	**±**	**+**	**+**	**+**	**+**	**+**	**+**	**+**	**+**	**±**	**-**	17+/2±	29
Curtis Breslin & Pole, 2009 [50]	**+**	**+**	**+**	**+**	**±**	**+**	**+**	**+**	**-**	**+**	**+**	**+**	**+**	**+**	**+**	**+**	**-**	**+**	**+**	**+**	**-**	**-**	17+/1±	5
Witte et al., 1998 [51]	**+**	**+**	**+**	**±**	**+**	**+**	**+**	**+**	**+**	**+**	**+**	**-**	**+**	**+**	**+**	**+**	**-**	**+**	**-**	**+**	**-**	**-**	16+/1±	7
Vogel & Holt, 2003 [52]	**+**	**+**	**+**	**+**	**+**	**+**	**+**	**+**	**-**	**+**	**+**	**+**	**±**	**±**	**+**	**+**	**-**	**+**	**-**	**±**	**+**	**+**	16+/3±	4
Taylor & Walter, 2003 [53]	**+**	**+**	**+**	**-**	**+**	**±**	**+**	**+**	**-**	**-**	**+**	**+**	**-**	**-**	**+**	**+**	**-**	**+**	**+**	**-**	**+**	**+**	14+/1±	4
Millward et al., 2005 [54]	**+**	**+**	**+**	**±**	**-**	**-**	**+**	**+**	**-**	**±**	**±**	**+**	**+**	**+**	**+**	**+**	**-**	**+**	**+**	**+**	**+**	**-**	14+/3±	6
Ingesson, 2007 [55]	**+**	**+**	**±**	**-**	**±**	**+**	**+**	**±**	**-**	**+**	**±**	**+**	**+**	**+**	**+**	**+**	**-**	**+**	**+**	**+**	**-**	**-**	13+/1±	7
Morris & Turnbull, 2007 [56]	**+**	**+**	**+**	**±**	**+**	**+**	**±**	**-**	**-**	**+**	**±**	**+**	**±**	**+**	**+**	**+**	**-**	**+**	**+**	**+**	**-**	**-**	13+/4±	51
Madaus et al., 2002 [57]	**+**	**+**	**±**	**±**	**+**	**+**	**+**	**+**	**-**	**+**	**-**	**-**	**±**	**+**	**+**	**+**	**+**	**+**	**±**	**-**	**±**	**-**	12+/5±	40
Schulte-Körne et al., 2003 [58]	**+**	**+**	**+**	**-**	**+**	**+**	**+**	**+**	**-**	**±**	**-**	**-**	**±**	**+**	**+**	**+**	**-**	**+**	**±**	**-**	**-**	**+**	12+/3±	3
Chapman et al., 2003 [59]	**+**	**+**	**+**	**+**	**-**	**+**	**±**	**±**	**-**	**+**	**-**	**-**	**+**	**±**	**+**	**+**	**±**	**+**	**-**	**+**	**-**	**-**	11+/4±	8
Magajna et al., 2003 [60]	**+**	**+**	**+**	**±**	**±**	**+**	**+**	**+**	**-**	**+**	**-**	**-**	**-**	**+**	**-**	**+**	**-**	**-**	**-**	**-**	**±**	**+**	10+/3±	6

Legenda: 1. **Title and abstract**; **Introduction:** 2. Background/rationale; 3. Objectives; **Methods:** 4. Study design; 5. Setting; 6. Participants; 7. Variables; 8. Data sources/measurement; 9. Bias; 10. Study size; 11. Quantitative variables; 12. Statistical methods; **Results:** 13. Participants; 14. Descriptive data; 15. Outcome data; 16. Main results; 17. Other analyses; **Discussion:** 18. Key results; 19. Limitations; 20. Interpretation; 21. Generalisability; **Other information:** 22. Funding

(‘+’ = present; ‘-‘ = not present; ‘±’ = insufficiently described).

A study should have a minimum of 15 plusses and three ‘±’. A column has been added to Table 2 for the number of factors found in each study. Of the 16 quantitative studies, 8 reached that threshold.

We measured interrater reliability at the level of the criteria by calculating a Cohen’s Kappa. For the quantitative studies, the Kappa was 0.66.

### Main characteristics of the quantitative studies

The quantitative studies are characterized in terms of study design and setting, aim of the study, method for data collection, statistical analysis, description and number of participants, and outcome measures in Additional file 2.

In three studies, data from the International Adult Literacy Survey (IALS) were used [52,59,60]. Curtis, Breslin, and Pole [50] used the data from the Canadian Community Health Survey of 2003. The remaining studies used original data in a cross-sectional survey design without control groups, except for that of Dickinson and Verbeek [47], who used a control group from the National Longitudinal Survey of Youth, and that of Witte et al. [51], who used controls matched by gender, major, degree, and graduation year. Rojewski [48] and Schulte-Körne et al. [58] performed longitudinal studies over 2 and 20 years, respectively. Further, a (small) survey-based exploration was executed in two studies [54,56].

With regard to the number of participants, the study of Curtis, Breslin, and Pole [50], with 14,379 participants, is an outlier. The sample size in the remaining studies ranged from 29 [58] to 805 [52]. One study does not mention the distribution of gender. In the other 15 studies, 9027 men (53%) and 7952 women (47%) took part; the age range is hard to determine because several studies only give the mean age and standard deviation. Both educational and employment status diverged strongly over the studies. Of the 17,673 participants, 17,323 are employed in a wide range of occupations; 291 are unemployed; and the employment status of 59 individuals is unknown. The setting from which the participants were recruited also varied strongly: from universities and special education institutions to dyslexia clinics. The outcome measures all have some relation to work but vary from job satisfaction, salary, occupational aspirations, occupation choice, work injuries, and employment self-efficacy to self-disclosure.

### Data extraction

Table 3 describes how the factors extracted from all studies fit into the ICF scheme, in the order of the categories mentioned in Figure 1. In all studies the work-related disease/disorder is called learning disability or dyslexia, not otherwise specified. The studies with both indicators were included for reasons mentioned in the Methods section. For this paper, the findings from the studies were scaled to the second level of the ICF categories, to avoid unreadable tables. On the first level, the ICF divides the main domains into chapters that are itemized at a second level. Separate columns were created in Table 3 for qualitative and quantitative studies. The studies that are in bold are those that meet the minimum level of the quality criteria. Four qualitative and eight quantitative studies did not reach that threshold. This leaves 21 studies (13 qualitative and 8 quantitative) on which to calculate the manifest frequency effect size.

**Table 3:** The factors from all studies coded on the two-level classifications of ICF

**2**^**nd **^**level ICF**		**Factors**		**Qualitative studies**	**Quantitative studies**
**Functions & structures**					
**b1 Mental functions**					
b114	Orientation functions			**36**^**-**^, **37**^**-**^	
b126	Temperament and personality functions	Adaptability		**34**^**+**^, **39**^**+**^	
		Optimistic			55
		Perseverance/persistence		**28**^**+**^, **29**^**+**^, **31**^**+**^, **35**^**+ **^**36**^**+**^, 42^+^	**46**^**+**^, 57^+^
		Personal characteristics			**47**
		Uncertain/insecure		**28**^**-**^, **29**^**-**^	
b140	Attention functions			**30**^**-**^, **34**^**-**^, **36**^**-**^	
b144	Memory functions			**31**^**-**^, **34**^**+**^, **36**^**- **^**37**^**-**^, 43^-^	56^-^
		Remembering instructions		42	
		Remembering names		42	
b152	Emotional functions	Amount of passion		**39**	
		Anxiety		**28**^**-**^, **37**^**-**^, **40**^**-**^	
		Aggressive feelings		**28**	
		Concern for patient safety			56
		Confusion/anger		**29**^**-**^, **30**^**-**^, **32**^**-**^, **37**^**-**^, 42^-^	
		Depression		**37**	
		Discouragement		**32**	
		Emotional problems		**28**	
		Fear of being stigmatized		**40**^**-**^, 42^-^, 43^-^	
		Fear of exposure		**37**^**-**^, 43^-^	
		Fear of failure		**28**^**-**^	56^-^
		Feelings of accomplishment		43^-^	**49**^**+**^
		Feeling of being different		**28**^**-**^, **37**^**-**^, 43^-^	
		Feeling of inadequacy		**34**^**-**^, 42^-^	
		Feeling of presenting a false impression (impostor phenomenon)		**37**	
		Frustration		**28**^**-**^, **30**^**-**^, **32**^**-**^**37**^**-**^	
		Guilt/embarrassment		**34**^**-**^, **37**^**-**^	55^-^, 56^-^
		Inferiority feelings		**28**^**-**^, **31**^**-**^, **37**^**-**^	56^-^
		Sense of strength		**29**^**+**^, **31**^**+**^, **35**^**+**^	
		Shame		**34**^**-**^, **37**^**-**^**,** 43^-^	
b156	Perception			**29**^**+**^, **37**^**+**^	
b160	Thought functions			**29**^**+**^, **34**^**+**^, **35**^**-**^, **36**^**+**^	56^+^
b164	Higher-level cognitive functions	Executive functions		**35**	
		Holistic/visual thinking		**31**^**+**^, **35**^**+**^	
		Increased creativity		**29**^**+**^, **36**^**+**^, **37**^**+**^	
		Information processing			**49**^**-**^, 57^-^
		Organization and planning		**29**^**-**^, **31**^**+**^, **36**^**+**^, **37**^**-**^, **39**^**-**^	**49**^**+**^, 56^-^, 57^-^
		Thinking outside the box		**29**	
b167	Mental functions of language	Word retrieval		**37**	
b180	Experience of self and time functions	Bothered by being different		**36**	
		Depersonalize LD		**36**	
		Disability self-awareness		**30**^**+**^, **35**^**+**^, **36**^**+**^	
		Labeling LD as a kind of self-mockery		**39**	
		Negative self-perceptions		**30**^**-**^, **34**^**-**^, **37**^**-**^	
		Not seeing themselves as having a disability		**30**^**+**^, **40**^**+**^	
		Self-conscious		**40**	
**Activities (d1- d6) and participation (d7- d9)**					
**d1 Learning and applying knowledge**					
d115	Listening			**36**	
d155	Acquiring skills	Analytical skills		**36**^**+**^	57^-^
		Developing coping strategies		**40**	
		Developing different learning styles		42	
		Learning a new skill		42°, 44^+^	
		Learning operating instrument names		42	
		Practical skills		**31**^**+**^, **35**^**+**^	
d160	Focusing attention			**31**^**+**^, **37**^**+**^	
d166	Reading			**28**^**-**^, **29**^**-**^, **30**^**-**^, **31**^**-**^, **35**^**-**^, **36**^**-**^, **37**^**-**^, **40**^**-**^, 42^-^, 43^-^	**45°**, 54°, 55^-^, 56^-^, 57^-^, 60^-^
d170	Writing (spelling)			**28**^**-**^, **29**^**-**^, **30**^**-**^, **31**^**-**^, **34**^**-**^, **35**^**-**^, **36**^**-**^, **39**^**-**^, **40**^**-**^, 42^-^, 43^-^	**49**^**-**^, 54^-^, 55^-^, 56^-^, 57^-^, 60^-^
		Writing reports to work		**33**^**-**^, 41^-^, 42^-^	
d172	Calculating			**36**^**+**^, 42^-^, 43^-^	56^-^, 60^-^
d175	Solving problems			**31**^**+**^, **34**^**+**^, **36**^**+**^, **37**^**+**^	57^+^
**d2 General tasks and demands**					
d210	Undertaking a single task			**37**	
d220	Undertaking multiple tasks	Correcting mistakes			**52**
		Gathering information		**36**°	56^-^
		Giving lectures/presentations/training sessions		**31**^**+**^, **35**^**-**^, 42^-^	
		Meetings		42^-^	56^-^
		Representing a group		42	
d230	Carrying out daily routine			**28**^**±**^, **36**^**±**^, **37**^**±**^, **40**^**±**^, 42^-^, 43^±^	
d240	Handling stress and other responsibilities			**36**	
		Handling responsibilities		**28**	
**d3 Communication**					
d310	Communicating with – receiving – spoken messages			**30**^**-**^, **36**^**±**^	
d315	Communicating with – receiving nonverbal messages	Receiving body gestures		**35**	
d325	Communicating with – receiving – written messages			**30**^**-**^, **36**^**-**^, **37**^**-**^	**49**^**-**^, 57^-^
d330	Speaking			**28**^**-**^, **31**^**+**^, **35**^**+**^, **36**^**±**^	56^-^
		Speaking in public			56
d335	Producing nonverbalmessages	Using drawings		**31**^**+**^, **35**^**+**^	
d345	Writing messages	Completing a literacy exercise		**40**	
		Filling in application forms		**30**^**-**^, **40**^**-**^, 42^-^	60^-^
		Keeping records		42^+^	56^-^
		Note taking		**30**^**-**^, **36**^**+**^, 42^-^	
		Taking telephone messages		42	
d355	Discussion	Discussing (LD with employer/problems with colleagues)		**30**^**-**^, **31**^**+**^, **34°**, **35**^**+**^	
d 360	Using communication devices and techniques	Dialing a phone		**36**	
		Multitasking (on a computer)		**36**	
		Using computer software/skills		**28**^**+**^, **35**^**+**^, **36**^**+**^, **40**^**+**^	57^-^
**d6 Domestic life**					
d660	Assisting others	Helping students		**31**^**+**^, **32**^**+**^, **35**^**+**^	
		Motivate students		**31**^**+**^, **32**^**+**^, **35**^**+**^	
		Validate students feelings		**31**^**+**^, **32**^**+**^, **35**^**+**^	
**d7 Interpersonal interactions and relationships**					
d710	Basic interpersonal interactions			**32**	
		Using social skills		**31**^**+**^, **35**^**+**^	**46**^**+**^
d720	Complex interpersonal interactions	Forming relationships		**28**^**-**^, **36**^**-**^	
d740	Formal relationships	Relationships with clients		41	
**d8 Major life areas**					
d845	Acquiring, keeping and terminating a job	Ability to maintain the career		**29**^**-**^, **40**^**-**^	56^-^
		Demotion or dismissal		**37**^**-**^, **40**^**-**^, 43^-^	56^-^
		Meeting their goals		**32**^**+**^, 42^+^	
		Occupation choice		42^-^	**46°**, 53^-^, 56^-^, 58^-^, 59^-^
		Securing his current position		**29**^**-**^, **34**^**+**^, **40**^**-**^	**46**^**-**^, **49°**, 57^-^
		Seeking employment		**40**	
		Skills for performing the job		43	
		Survive within the workplace		**40**	
		Terminating a job		**34**^**-**^, 43^-^	
		Working much harder		**28**^**-**^, **31**^**-**^, **35**^**-**^, **36**^**-**^	
d850	Remunerative employment	Achievement/accomplishment		**28**^**-**^, **29**^**±**^, **31**^**-**^ 42^±^, 43^-^	
		Continuous changing of employment		**40**	
		Employment roles		**40**	
		Employment in unskilled/semiskilled occupations		**28**^**-**^, **40**^**-**^	**50**^**-**^, 60^+^
		Employment in white-collar/blue-collar jobs			**45°**, **52**^**+**^, 53^+^, 55^+^
		Enabled to excel		**36**	
		Having a job/being employed		**28**^**+**^	**47**^**+**^, **51**^**+**^, 55^+^
		Having more jobs			**47**
		Job history		**28**	
		Job at the level of their training or higher		**28**^**+**^, **36**^**+**^	
		Job performance		**33**^**+**^, **34**^**+**^, 43^-^	
		Limited employment opportunities		43^-^	**47**^**-**^, 55^-^
		Part-time employment		**28**^**+**^, **29**^**+**^	55^-^
		Permanently employed			55^+^, 60^-^
		Quality of products and processes		41	
		Unemployed		**28**^**-**^, **40**^**-**^	**48**^**-**^, **52**^**-**^, 59^-^
		Workforce abilities			**47**
**Work-related environmental factors: terms of employment**					
		Access to degree courses		42^-^	56^+^
		Attendance		**33**	
		Change in job description/title		**34**	
		Current (annual) salary			**46°**, **49**^**+**^**°**, **51**^**-**^, **52**^**-**^, 59°
		Extra study leave		42	
		Extra time		**34**^**+**^	56^+^, 57^+^
		Flexible schedule		**33**^**+**^, 41^+^	
		Incentives and bonuses		**34**	
		Income derived from employment			59
		On-the-job training agreement		**31**^**+**^, **33**^**+**^	
		Promotion/Job advancement		**30**^**+**^, **33**^**+**^, **34**^**+**^, 42^+^	**51**^**-**^
		Tenure used as productivity signal		**30**^**+**^	**47**^**+**^
		Tenure with current employer		**30**^**+**^	**47**^**+**^
		Wage -gap (with non-LD)			**47**^**-**^, **51°**, **52**^**+**^
		Wage increase/decrease		**33**^**+**^, **34**^**+**^	**47**^**+**^
		Working from home		41	
		Working at off hours late in the day or night		**36**^**+**^, 41^+^, 42^-^	**46**^**+**^, **49**^**+**^, **50**^**+**^ 56^+^, 57^+^
**Work-related environmental factors: social relationships at work**					
**e3 Support and relationships**					
e315/e320	Extended family/friends	Support from family/friends		**28**^**+**^, **29**^**+**^, **32**^**+**^, **33**^**+**^, **34**^**+**^, **35**^**+**^, **39**^**+**^	55^+^
e325	Acquaintances, peers, colleagues, neighbors and community members	Being loners/isolated in the job		**37**	
		Control/check by others		**28**^**+**^, **31**^**+**^, **35**^**+**^, **36**^**+**^, **37**^**+**^, 42^+^	56^+^
		Encouragement			56
		Help from colleagues/co-worker assistance		**28**^**+**^, **30**^**-**^, **31**^**+ **^**34**^**+**^, **35**^**+**^, **37**^**+**^, 41^+^, 42^+^	**46**^**+**^, **49**^**+**^, 56^+^
		Joint goal planning		44	
		Joint working		**35**^**+**^, 44^+^	
		Peer pressure		**36**	
		Performance feedback		**37**	
		Secretary dependent		**36**	
		Social relationships with co-workers (trust)		**31**^**+**^, **35**^**+**^, **37**^**-**^	56^+^, 57^±^
		Support in the workplace		**29**^**+**^, **30**^**-**^, **34**^**+**^, **35**^**+**^**,** 42^+^	56^+^
		Workforce morale		42	
e330	People in position of authority	Employer assistance-support		**30**^**-**^, **34**^**+**^, **38**^**+**^, 42^-^	**47**^**+**^, **49**^**+**^, 56^+^, 57^+^
		Role of employer-supervisor		**30**^**+**^, **31**^**+**^, **37**^**+**^	**51**^**-**^, 57°
		Support of rehabilitation staff		**33**	
**e4 Attitudes**					
e425	Individual attitudes of acquaintances, peers, colleagues, neighbors and community members	Appreciation of/consideration to mental disabilities		41	
		Attitudinal barriers		41^-^, 42^-^	
		Awareness/detection of the disability		**34**^**-**^, **36**°, **40**^**-**^, 41^+^, 42^-^	
		Discrimination		**40**^**-**^, 41^-^, 43^-^	**47**^**-**^
		Interest in, acceptance / understanding of dyslexia		**28**^**-**^, **34**^**-**^, 41^-^, 42^-^, 43^-^	56^-^, 57^-^
		Labeling people with LD as lazy, slow, dumb or stupid		**22**^**-**^, **39**^**-**^, 41^-^, 42^-^	
		LD makes me important		**34**	
		LD as a tool to address misconceptions about LD		**32**^**+**^	57^+^
		Reactions of co-workers		**28**°, **31**^**- **^**32**^**+**^, **34**^**±**^, **35**^**-**^, **36**^**-**^, **39**°, **40**^**-**^, 41^-^, 42^-^, 43^-^	56^±^
		Stigmatization by colleagues		**40**^**-**^**,** 42^-^, 43^-^	
e430	Individual attitudes of people in position of authority	Acceptance of dyslexia by employer		**34**^**+**^, **36**^**+**^, 43^+^	56^-^
		Detection/awareness of the disability		**34**^**-**^, **36**^**-**^, **40**^**-**^, 41^-^, 42^-^	**47**°
		Employer attitudes and knowledge		**30**^**+**^, **38**^**-**^, 42^-^	
		High expectations of people with LD		**32**^**+**^, 43^-^	
		Judgment of work on disability		**28**	
		Negative responses from employer		**31**^**-**^, **34**^**-**^, **40**^**-**^	
		Stigmatization by employer		**40**	
**Work-related environmental factors: task content**					
		Benefits of current position		**33**	
		Challenging students to achieve their potential		**31**^**+**^, **35**^**+**^	
		Control over their work		**31**^**+**^, **36**^**+**^,	
		Demands involving reading or writing			53^-^, 55^-^, 58^-^
		Demands of the job		**33**^**-**^, **37**^**-**^	**46**^**+**^
		Finding a niche		**29**^**+**^, **36**^**+**^	
		Increase of independence		**36**	
		Job level		**28**	
		More responsibility		**31**^**+**^, **30**^**+**^, **33**^**+**^, **34**^**+**^	
		Much information in a constant stream			56
		Multitasking is required			56
		Prioritizing tasks			**46**^**+**^, **49**^**+**^, 57^+^
		Productivity demands		**33**	
		Self-regulation			**46**^**+**^, **49**^**+**^
		Taking work home		41^+^, 42^-^	
		Working under pressure		42	
		Workload, pressure, more stress		**29**^**-**^, **34**^**-**^, 41^-^, 42^-^, 43^+^	56^-^
		To work by myself		**30**^**+**^, **37**^**+**^	
**Work-related environmental factors: working conditions**					
e125	Products and technology for communication	Assistive technology (like a dictaphone, internet, navigation software, watch, microcassette)		**32**^**+**^, **34**^**+**^, **35**^**+**^, **36**^**+**^, **40**^**+**^, 42^+^, 43^+^	**46**^**+**^, 57^+^
		Clear signs		42	
		Computer spelling and grammar programs		**28**^**+**^, **35**^**+**^, **36**^**+**^, 42^+^	56^+^
		Simple words and pictures		42	
		Technology interferes with work/Experiment with technology		**36**	
		Visual aids		**31**	
		Voice-mail		**36**	
e135	Products and technology for employment	Accommodations on the job		**29**^**-**^, **30**^**-**^, **32**^**+**^, **34**^**+**^, **36**^**+**^, **38**^**-**^, **40**^**-**^**,** 42^+^, 43^-^, 44	**46**^**+**^, **49**^**-**^, 57^+^°
e1650	Financial assets	Budget for an assistant		**28**^**+**^, **34**^**+**^	
		Money		**40**^**-**^, 41^-^	
	Other working conditions	Accessibility of the office environment		41	
		Changing/improving the work environment		42	
		Finding a quiet/personal work environment		**35**^**+**^	**46**^**+**^
		Hearing the words spoken aloud		**36**	
		A mentor		**33**^**+**^, **34**^**+**^	
		Time		**31**^**-**^, **34**^**+**^, **35**^**-**^, **37**^**-**^, 41^-^, 42^-^	
		Using proofreaders		**35**^**+**^, **36**^**+**^, 43^+^	**46**^**+**^, 57^+^
**Other work-related environmental factors**					
		Centrality of LD in his career		**39**	
		Critical knowledge and experiences on LD of employers		**38**	
		Disabling barriers		**40**	
		Information about (the impact of) dyslexia for employers		**30**^**-**^, **38**^**-**^	
		Job shadows		**33**	
		Job visits		**33**	
		School-to-work program		**33**	
		Training sessions regarding disability		41	
		Vocational rehabilitation		**33**	
		Work-related injury risk			**50**
**Other environmental factors**					
e550	Legal services, systems and policies	Certifying disability		41	
		Disability Discrimination Act		41	
		Diversity agenda		41	
		Equality issues		41	
		Knowledge of the ADA		**30**	
		Legislation on adults with LD		**34**^**+**^, 41^+^	
e570	Social security services, systems and policies	Income from government benefits/resources			59
**Personal factors**					
---	General personal data:	Age			**45°**, **46°**, **49°**, **52°**, 60°
	Socio-demographic factors				
		Gender			**45**^**+**^, **46°**, **47**^**-**^**48**^**-**^, **49°**, **50**^**+**^, **52**°. 54°, 60°
		Language			54
		Socio-economic status		**39**^**+**^, **40**^**-**^	**48**^**+**^
		Standard/level of education		41°	**45°**, **48**^**+**^, 59^+^, 60^+^
---	General ‘mental’ personal factors	Aggravation		**30**	
		Common sense			56
		Confidence		42^+^	56^+^
		Creative		**31**^**+**^, **37**^**+**^	56^+^
		Demoralized		**40**	
		Desire to help others		**31**^**+**^, **37**^**+**^	
		Detail oriented		**36**	
		Diligent			56
		Empathic		**31**^**+**^	56^+^, 57^+^
		Humorous		**35**^**+**^	56^+^
		Innovative		**31**^**+**^, **37**^**+**^	
		Learning/Coping strategies:		**28**^**+**^, **29**^**+**^, **31**^**+**^, **37**^**+**^, **40**^**+**^, 41^+^, 42^+^	**46**^**+**^, **47**^**+**^, **49**^**+**^, 56^+^
			Adjusted to the disability	43	
			Ask caller to speak slowly	42	
			Authoritative language	**39**	
			Avoid my weaknesses	**36**	
			Avoidance	**28**^**-**^, **35**^**-**^, **36**^**+**^	
			Aware of limitations	**35**	
			Be aware of problem words	42	
			Bluffing	**39**	
			Camouflage	**28**^**-**^, **40**^**-**^	
			Compensatory skills	**36**^**+**^, **39**^**+**^, 43^+^	**47**^**+**^
			Develop own shorthand	42	
			Disclosing weaknesses	**31**	
			Distancing himself from others	**29**^**-**^, **39**^**-**^	
			Establish themselves as intelligent	**40**	
			Excessive amount of energy in work	**28**	
			Exposing own difficulties	**31**	
			Get writings/forms checked	**35**^**+**^, 42^+^	
			Give colour coded feedback	**35**	
			Hiding the LD	**35**^**-**^, **37**^**-**^, **40**^**-**^	
			Leave and come back later, when losing concentration	42	
			Make careful mental and concrete preparations	**35**	
			Make lists or leave notes for myself		56
			Make people write things down what they have said	**35**^**+**^, **36**^**+**^	
			Mnemonics	**35**	
			Other practical solutions	**28**	
			Overcompensation	**28**	
			Paper and pencil in pocket	42	
			Pattern of overachieving	**29**	
			Physical activities to recall ideas	**35**	
			Read back messages to caller	42	
			Read novels to experience different types of writing	42	
			Repeat names many times	42	
			Repression	**28**	
			Tricks	**28**	
			Using prompts to recall topics	**35**	
			Use set phrases	42	
			Visualization techniques	**35**	
			Write things out in rough	42	
		Literacy level		**40**^**-**^	54^+^, 59^+^, 60^+^
		Openness		**28**^**±**^, **30**^**-**^, **31**^**+**^, 42^-^, 43^-^	
		Patience		**36**	
		Personal problems		**28**	
		Pride		**29**	
		Satisfaction with life		**28**	
		Secretive		43	
		Sensitive to emotional experiences of others		**29**^**+**^, **31**^**+**^, **36**^**+**^, **37**^**+**^	
		Self-advocacy		**30**^**±**^, **38**^**+**^, 44^+^	
		Self-control		**28**	
		Self-disclosure		**30**^**-**^, **32**^**+**^, **34**^**+**^,**35**^**+**^, **37°**, **38**^**-**^, **39°, 40**^**-**^, 43	**46**^**-**^, **49**^**0**-^, 56^+^, 57
		Self-efficacy		**35**^**+**^	**46**^**+**^, **49**^**+**^
		Self-empowerment		**39**	
		Self-esteem		**29**^**+**^, **31**^**+**^, **32**^**+**^, **35**^**+**^, **36**^**+**^, **39**^**+**^, 42^+^	**48**^**+**^
		Self-promotion		**30**	
		Self-reliant		**36**	
		Self-sufficient		**36**	
		Social person		**36**	
		Stress avoidance		**28**	
		Stress-experience/being stressed		**28**^**-**^, **29**^**-**^, **34**^**-**^, **35**^**-**^, **36**^**-**^, **37**^**-**^, 42^-^	
		Stubborn			57
		Tolerant			56
		Visual		**31**^**+**^, **35**^**+**^	
---	Health related personal factors	Accepting the LD/dyslexia		**28**	
		Experiences with LD/dyslexia		**29**^**-**^, **37**^**-**^	
		Impact of the LD/dyslexia		**28**^**-**^, **36**^**-**^, **37°, 40°**, 41^-^, 42^-^, 43^-^	**46**^**-**^, **49**^**0**-^, 55^-^, 56^-^. 57^-^
		Locus of control			**48**
		Management of the LD/dyslexia		**40**	
---	Work related personal factors	Collaborative/co-operative		**31**	
		Commitment		**33**	
		Compliance		**38**	
		Determination		**31**^**+**^, **35**^**+**^, **36**^**+**^, 42^+^	56^+^
		Focus		**33**	
		Force of will		**39**	
		Goal driven		**33**	
		Independence		**29**^**+**^, **32**^**+**^, **36**^**+**^	**49**^**+**^
		Job/career satisfaction		**28**^**0**+^, **36**^**+**^, 43^+^	**46**^**+**^, **49**^**+**^, **51**^**-**^
		Prestige aspirations		**31**^**+**^	**48**^**+**^
		Proactive		**31**^**+**^	
		Productivity/worker characteristics			**47**
		Successful		**29**^**+**^, **31**^**+**^, **32**^**+**^, **36**^**+**^, 41^+^	
		Tenacity		**34**	
		Underchallenged		**29**	
		Work ethic		**29**	

The studies which are in bold are the studies that meet the minimum level of the quality criteria. (In each category the findings are in alphabetical order).

In Table 3 the consistency of each factor (barrier or facilitator) that is mentioned in more than one study has been made visible by using the signs + and − for positive and negative, respectively; ± when the interpretations of the factor by the interviewed participants in a study were opposite; and finally 0 when a factor was mentioned but without an explicit interpretation by the participant.

In Table 4 the manifest frequency effect sizes (MFES) of the factors in the different ICF- categories are summarized.

**Table 4:** The manifest frequency effect size for each ICF-category

**ICF-category**	**MFES**
Mental functions	67%
Activities	71%
Participation	86%
Terms of employment	52%
Social relationships at work	81%
Task content	52%
Working conditions	67%
Other work-related environmental factors	29%
Other environmental factors	10%
Personal factors	100%

Within the category ‘mental functions’, the ‘emotional functions’ were frequently mentioned: 11 of the 21 studies with sufficient quality mention factors coded as emotional functions, which amounts to a manifest frequency effect size of 52%. Of the 20 emotions mentioned, only 3 have a positive connotation: ‘amount of passion’ [39], ‘feelings of accomplishment’ [49], and ‘sense of strength’ [29,31,35]. This is consistent with the experience of self: in three studies [30,34,37] negative self-perceptions were mentioned by the participants.

The activities ‘reading’ (d166), ‘writing/spelling’ (d170), and calculating (d172) were mentioned in 14 of the 21 studies (effect size 67%). In these 14 studies the activities were always discussed in relation to work, and mostly within the framework of the task content or social relationships at work. The interpretation of ‘reading’ or ‘writing’ was consistently negative, while ‘calculating’ was mentioned as a strength in one study [36]. ‘Solving problems’, mentioned in three studies, was always seen as a strength. Four studies mentioned the effects of LD on carrying out daily routines, but the interpretation was inconsistent.

Within the category ‘participation’, 11 of the 21 studies mentioned 'acquiring, keeping and terminating a job’ (d845) (effect size 52%). Notably, ‘securing his/her current position’ (d8451) was mentioned in five studies (24%) with inconsistent interpretations. In 13 studies ‘remunerative employment’ (d850) was mentioned (62%); seven studies mentioned having a job, or being employed in unskilled/semi-skilled occupations or in a white- or blue-collar job (33%); also with inconsistent interpretations.

Among the various terms of employment, 'salary' (current annual salary, wage gap, or wage increase/decrease) was discussed in seven of the 21 studies (33%), with inconsistent interpretations. Four of them mentioned working at off hours, either late in the day or at night, which was consistently interpreted as positive. This factor corresponds with remarks about taking work home and putting an excessive amount of energy into one’s job in order to survive in the workplace.

The category of ‘social relationships at work’ also contains factors that occur frequently. Nine studies mentioned ‘support in the workplace/help from colleagues’ (e325) (43%), and five referred to ‘employer assistance or support’ (e330) (24%). Eight studies mentioned 'reactions of co-workers' (38%), with inconsistent interpretations.

Two factors within the category of ‘working conditions’, namely ‘products and technology for communication’ (e125) and ‘products and technology for employment’ (e135) were mentioned in eight (38%) and nine (43%) studies, respectively. Among the ‘products and technology for communication’, six studies mentioned assistive technology, such as a dictaphone, the Internet or navigator (28%), which was always interpreted positively. Among the ‘products and technology for employment’ nine mentioned accommodations on the job (43%), with various interpretations.

‘Gender’, which is a socio-demographic characteristic, was mentioned in seven studies (33%) as a predictive factor. It is seen as a factor in different work contexts: for reading practices [45]; for occupational aspirations [48]; for a wage gap [47]; for employment satisfaction [49]; and for work injuries [50].

Within the subcategory of ‘general ‘mental’ personal factors’ two factors occurred frequently: ‘learning/coping strategies’, mentioned in 8 studies (38%); and ‘self-disclosure’, mentioned in 10 (50%). 'Self-esteem,' a factor affected by the LD, was mentioned seven times (33%) in a positive way. 'Stress experience/being stressed' occurred six times (28%), consistently negative. Within the subcategory of ‘health-related personal factors’ the ‘impact of the dyslexia’ was discussed in six studies (28%), three times without and three times with a negative interpretation. And finally, within the subcategory of ‘work-related personal factors’, ‘job/career satisfaction’ was mentioned in five studies (24%), with inconsistent interpretations.

## Discussion

In this literature review, we found 318 factors associated with participation in work of employees with developmental dyslexia. The following factors were mentioned most: persistent difficulties in reading or writing/spelling; the participants'—mostly negative—feelings and emotions about dyslexia; or difficulty in acquiring a job and, once acquired, keeping it, be it a blue- or white-collar job. Furthermore, 'self-disclosure' or the ‘support’ of colleagues and employer,' mostly seen as positive factors, and the ‘attitudes’ about and reactions to disclosure among co-workers were frequently mentioned. The ‘use of assistive technology for communication’ positively influenced work participation, as did some accommodation in the workplace. ‘Job/career satisfaction’ was also mentioned as an important factor. At a personal level, the studies often mentioned the ‘impact of dyslexia’, mostly interpreted as a negative factor, and the acquired ‘coping and compensation strategies’, which were consistently positive factors. These most important factors cover a wide scope of items from the main domains of the ICF.

With respect to participation, ‘having a job’ or ‘being employed’ and ‘securing the current position’ seemed to be very important factors. That observation is consistent with results from the IALS studies (International Adult Literacy Survey) [52,59,60]. In these studies the choice of the measurement instruments is not particularly dyslexia-friendly, and results in a sample of highly competent adults with dyslexia. The IALS studies reveal differences between the self-reported learning disability group (SRLD group) of highly competent participants and the non-SRLD group in terms of finding and maintaining a job. These differences only apply to the highly competent sample that participated in these IALS studies. For most people with dyslexia—as for most employees—having a job is so important that they will try to keep it.

Regarding the terms of employment, a ‘wage gap’ between employees with and without a learning disability has been confirmed by Dickinson and Verbeek [47]. However, this gap cannot be fully explained by differences in productivity characteristics due to the presence of a learning disability. Differences in annual salary are also reported in the IALS studies. Decision latitude and work autonomy—to be attained by means of extra time, a flexible schedule, or working from home—are considered important terms of employment. Self-regulation and control over the work—to be gained by taking work home and prioritizing tasks—have a positive effect on one’s focus, productivity, and motivation. This is consistent with a model that Gerber et al. presented in 1992 [61] for describing success in work for employees with DD. In this model, ‘control’ was the main factor in achieving success.

The authors reviewed here tended to diverge with respect to the occupational choices made by adults with DD. Some emphasized the choice of creative or administrative professions, others of more hand-crafted occupations. The studies underlying this review gave no consensus on whether employees with DD were more likely to be employed in white- or blue-collar jobs. In some studies participants indicated they were working at a level lower than their education.

A majority of the studies presented the perspective of the employee with dyslexia. One study also focused on the employers (Human Resource Managers) [41]. Teachers with DD form a special category [31,35,39]: having DD themselves, they seem to be role-models for their students. In their dual role, they build a special relationship with their students (especially those with dyslexia). In that relationship, the dyslexia serves a special function: as a tool to make them better professionals [39] and as a tool to challenge students to achieve their potential [31].

Although the majority of the findings are indicative of problems that have to be overcome, there are also many factors with a positive connotation, like being creative, solving problems, being persistent, and using dyslexia in educating dyslexic students. Nevertheless, in none of the studies do the included participants emphasize the strengths of dyslexia with respect to the company for which they work. A possible explanation can be found in research by Fitzgibbon and O’Connor [62] and also by Logan [16]. Their research suggested that the corporate environment is not conducive to people with dyslexia. A structured environment is very stressful for employees with DD. They prefer situations in which they themselves can control the variables [53] and where positive characteristics prevail. In order to create an environment in which they can flourish, many employees with DD start their own businesses and become entrepreneurs [16].

Most of the findings of this review do not have an exclusive relationship with dyslexia; they have a more general validity. For example, all employees thrive on support from their colleagues or employer; autonomy is important to every employee [63] and assistive technology is also relevant to workers with a chronic somatic disease, for instance [64]. But our focus is on employees with DD or a learning disability. For them, the significance of these factors is much greater than for a general healthy population.

Most of the studies approached dyslexia from a medical perspective: it is a disability that causes problems for the person who has it. Only one study [40] took a social approach: it is society that causes problems for the person with dyslexia by making demands that he/she can barely fulfill, and society is not willing to support the dyslexic adult in solving these problems.

### Strengths and limitations of the literature review

In this review we used the basic version of the ICF, enriched with the Van Dijk model [26] It was occasionally difficult to conceptualize some of the findings in accordance with the ICF. A third reviewer (YH) was consulted several times to discuss these classification problems.

To define the target population, we used the definition of learning disabilities from the National Joint Committee on Learning Disabilities [20]. This definition fit well with the ICF, but included a risk in the great heterogeneity of subjects in the studies for this review. That risk has been minimized by searching for the term ‘dyslexia’ in the Methods and Results sections. Nevertheless, the question remains of whether all factors are as relevant for the diverse groups of subjects. Furthermore, learning disabilities as an umbrella term has a higher prevalence than developmental dyslexia in a general population. The great range in prevalence figures is connected with the chosen definition.

A third problem was the interpretation of the factors: ‘occupation choice’, for instance, was mentioned three times as being affected by the learning disability and, hence, interpreted negatively. There was some question of whether that negative interpretation was really meant by the participants. For ‘employer assistance/support’, the factor is interpreted negatively in two studies because of a lack of that support. If that support is missing, the factor has a negative impact on work participation. Finally, in two studies ‘gender’ is labeled negatively, because it has a negative impact on prestige aspirations and employment status. Thus, for a correct interpretation of factors, sometimes more knowledge is needed about the underlying mechanisms and context.

A fourth problem was the range of meanings each factor can have. For instance, the factor ‘reading’ covers a broad range of meanings, such as the speed of reading, the reading performance, the understanding of what was read, and retention of the information read. So, the content of a factor may cause differences in interpretation. In this review the range of meanings within factors was not taken into account.

The ICF combines the health status (disease/disorder and problems in functioning) of an employee with DD and contextual factors (environmental and personal factors). For the research question of our study, the ICF scheme, when expanded with work-related environmental concepts of the Van Dijk model, seemed to give us a good grip on the categorization and positioning of factors.

In this review a distinction was made between qualitative and quantitative studies. This was done in the expectation of finding factors of a different nature in both categories. Looking at Table 3, this hypothesis is partly confirmed.

There is more to the subject, however, than can be conveyed by merely summarizing all of the relevant factors. It would be interesting to know whether the factors we have found influence one another and, if so, then what kind of relation they have and how that relation is to be interpreted. For instance: What is the relation between negative feelings and job satisfaction? What is the influence of access to assistive technology for communication on securing one’s job? Do persistent problems in reading or writing/spelling have an impact on one’s occupational choice? With the ICF classification system, we can describe the factors we found, but we cannot retrieve information about the underlying mechanisms, which is needed to answer that kind of questions.

A good example of such an underlying mechanism is the model of Gerber et al. [61]. This model explains the successful vocational functioning of adults with learning disabilities. In this model, success depends on the level of control over the situation. Control is determined by a dynamic interaction between the internal decisions and external manifestations of an individual. Leather et al. [4] provided quantitative support for this model and enriched it with planning skills and meta-cognitive awareness. Our main results support the Gerber-Leather model. One of the key issues in obtaining success is the capability of reframing: the re-interpretation of the learning disability in a more positive manner. In our review, most of the feelings and emotions about dyslexia were negative as was the impact of dyslexia in daily life. These feelings are a barrier for obtaining success and need to be reframed. The factor ‘self-disclosure’ fits in the category of ‘internal decisions’ in the model, but is not mentioned as such and is hard to classify in one of the subcategories of ‘reframing’, ‘desire’, or ‘goal oriented.’ But ‘self-disclosure’ is part of the meta-cognition that Leather et al. add to the model. The support of colleagues can be classified in the ‘social ecologies’ in the model. The acquired coping and compensation strategies fit in the ‘learned creativity’ category.

The Gerber-Leather model was constructed from the perspective of personal input in the work environment. But there is also a work input: the broader context of work with or without the possibility of using assistive technology or asking for accommodations. In this work context the attitude of employer and colleagues towards working with a disability is also of great importance. It would be worthwhile to investigate more closely the intersection of the personal and work input, in order to determine all the factors that influence the quality of the working life of adults with DD.

### Suggestions for further research

The ICF provided a clear framework for classifying the results. Its detailed structure also gives insight into areas that are not covered in the studies included in the sample. Visual and auditory processing is not mentioned by the participants in the qualitative studies, although this issue has been known in the literature since the early 1990s [65,66]. Auditory processing deficits were detected by Amitay et al. [67]. Nor is reasoning strategy as a higher-level cognitive function mentioned in the sample. Yet in 2003, Bacon et al. [68] presented evidence of individual differences in reasoning strategies among people with and without dyslexia.

Furthermore, the absence of references to motor impairment, balance, and pointing tasks as well as to bimanual coordination, is noteworthy. Motor impairments in people with dyslexia have been known for a long time [69]. Balance problems were studied by Fawcett and Nicholson in the 1990s [70] and more recently by Needle et al. [71]. Ever since the early publications of Orton [72,73]. Bimanual coordination and dyslexia have been an object of research in a stream of studies [74,75]. While these factors have not been explicitly related to employment, they might be. Nevertheless, they are not mentioned in relation to work in the studies underlying this review.

The emphasis in this review was on work participation of adults with DD. More explicit research would be advisable on the areas that remain underexposed in the categorization, carried out in accordance with the ICF. In particular, research is warranted on the relations between visual and auditory processing and reasoning strategies on the one hand, and the execution of tasks within the job on the other. The same applies to the relations among motor impairments, balance, and pointing tasks, on the one hand, and bimanual coordination and work, on the other.

This review yielded many factors influencing work participation. It would be very interesting to look more carefully into the relations among those factors and to consider the underlying mechanisms and context in which those factors are active. This can be done by performing qualitative in-depth interviews with employees with DD, their colleagues, and their supervisors. The findings in this review and the results of the in-depth interviews can be used in a deep analysis to formulate a model for work participation. Therefore it would be useful to have a more interpretative model that is also applicable to work. Mitra [76] and Welch Saleeby [77] have tried to understand a disability (like dyslexia) through the Capability Approach [78]. That approach distinguishes between a person’s capabilities (potential to achieve a set of actual opportunities) and functionings (achievements). For the validation of the numerous factors found in this review, it would be interesting to use the Capability Approach as a framework. Hypotheses could be formulated about the relations among the different factors. Once these relations have been clarified, they can be used to develop strategies or interventions to influence the quality of the working life of employees with developmental dyslexia.

## Conclusion

The adjective ‘developmental’ in the term ‘developmental dyslexia’ is salient. DD start small and initially affect the activities of writing/spelling and reading. But over the lifespan, DD affect more domains of human functioning: the personal and environmental domains and participation. And within each domain the impact of DD increases over the life course. In the context of work, all domains of functioning can be influenced by DD, as has been made visible in the main results of this review, by classifying the factors found in the literature in the ICF as enriched by the Van Dijk model. If that influence is negative, employees with DD seek support, compensation, or adaptation strategies: at a personal level by embracing several learning or coping strategies or by choosing self-disclosure; at an environmental level by asking for support, accommodations, or assistive technology; and at a societal level by aiming for legislation that protects their rights.

Noticeable also is the lack of a positive attitude toward DD, either by the adults with DD themselves—with the exception of teachers with DD—or by people in the environment. DD seldom are seen as a source of strength, creativity, or other positive competency with a great value to the company for which the employee with dyslexia is working.

## Competing interests

The authors declare that they have no competing interests.

## Authors’ contributions

All authors have substantially contributed to the conception and design of the review. JB, JE and JvdK performed and supervised the review procedure, data extraction and data classification and YH supervised the categorization of the factors in the International Classification of Functioning, Disability and Health (ICF). All authors cooperated in writing the manuscript, have read and approved the final manuscript, accept full responsibility for the design and the conduct of the review, had access to the data and approved the decision to publish.

## Pre-publication history

The pre-publication history for this paper can be accessed here:

http://www.biomedcentral.com/1471-2458/14/77/prepub

## Supplementary Material

Additional file 1 **Main characteristics of the qualitative studies **[28-44,79-97]**.** Click here for file

Additional file 2 **Main characteristics of the quantitative studies **[45-60,98-106]**.** Click here for file
