# Supplementary material for: Factors influencing work participation of adults with developmental dyslexia: a systematic review
Source: BMC Public Health. 2014 Jan 24;14:77. doi: 10.1186/1471-2458-14-77 (PMC3913008; doi:10.1186/1471-2458-14-77)
Supplement: Additional file 1 — Main characteristics of the qualitative studies [28-44,79-97]. [file 1471-2458-14-77-S1.doc]

Additional file 1. Main characteristics of the qualitative studies

| **Author(s)** | **Theoretical framework** | **Aim of the study** | **Method for data collection** | **Description and number of participants** |
| --- | --- | --- | --- | --- |
| Hellendoorn and Ruijssenaars, 2000 [28] | A qualitative, multiple case study design [79] | How do adults with dyslexia experience their disability, how did they grow up with it, and live and cope with it in their personal and working lives? How is their self-concept and how did the dyslexia influence their socio-emotional development? | Open, in-depth interview [80]. A topic checklist with tentative questions was developed. The interviews ranged from 100 to 245 minutes with an estimated mean of over 2 hours. | *Participants:* all were diagnosed with primary dyslexia, but had no other major disability  *Gender:* 12 men, 15 women (n=27)  *Range of age*: 20 – 39  *Education*: high school*,* nursing training, lower technical, domestic or administrative school, college or university  *Employment status:* 7 participants unemployed and 20 employed in different occupations |
| McNulty, 2003 [29] | The life story method of narrative analysis [81] | Understanding how the life stories can unfold, hold potential to help parents and professionals intervene in a more sensitive, supportive and effective manner. | One or two individual interviews with a total time between 3 and 5 hours. | *Participants:* were diagnosed with dyslexia prior to age 14  *Gender*: 8 men, 4 women (n=12)  *Range of age*: 25 – 45  *Education*: all completed high school.  *Employment status*: all were employed |
| Price et al., 2003 [30] | A collective case study design [82] | In essence, what are the issues for employees with learning disabilities in job acquisition, job advancement, self-disclosure and experiences with employer attitudes and beliefs? | Face-to-face interviews, ranging from 45 minutes to 2 hours in length. | *Participants:* were diagnosed with Learning Disabilities (LD)  *Gender*: 17 men, 8 women (n=25)  *Range of age*: 19 - 32  *Education*: all had average to above-average intelligence  *Employment status*: all worked in many different occupations |
| Burns and Bell, 2011 [31] | A combined theoretical framework of narrative research [83] and positioning theory [88] | What kind of narrative resources can be identified in interviews when teachers revealed their experiences of what it is like to be a teacher with dyslexia? How are the identified narrative resources utilized in the narrative construction of teacher identity? | Narrative interviews, lasting from an hour and a half to two hours. | *Participants:* teachers from England and Finland and were diagnosed with dyslexia  *Gender:* 5 men, 3 women (n=8)  *Range of age:* late 30s to late 50s  *Education:*  *Employment status:* all were employed in tertiary education from less than one year to over 30 years |
| Ferri et al., 2001 [32] | A qualitative multi-case study approach following a constant comparative method for analysis [84] | How do individuals who are currently teaching students with LD and who have received special education services during their school careers view the special education services they received? How do their past experiences with receiving special education influence their current practice? | In-depth, semi-structured interviews; two in the participant’s classrooms and one at a university. Each interview lasted approximately 1½ to 2 hours. | *Participants:* teachers with LD  *Gender:* 2 men, 1 woman (n=3)  *Range of age:* 28 - 29  *Education:* all had bachelor’s degrees and were taking graduate classes in special education  *Employment status:* all are teaching students with LD in resource rooms in public schools |
| Lindstrom and Benz, 2002 [33] | Case study methodology [79;85] | This study examines the factors that influence the career development process for young women with learning disabilities, entering the workforce. | In-depth open-ended interviews with the participant and key informants (parent, school staff member involved in the career preparation, vocational rehabilitation counselor, direct supervisor or manager on the job site). Interviews lasted 1 to 2 hours. | *Participants:* young women with LD  *Gender:* 6 young women (n=6)  *Range of age:*19 - 21  *Education:* all received special education services in a public high school and graduated from high school  *Employment status*: all were employed at least 30 hours per week |
| Gerber et al., 2004 [34] | A qualitative case study design [86;87] | To explore what differences, if any, there are between the US and Canadian workplaces for adults with LD. | Face-to-face interviews varied in length from 45 to 120 minutes. | *Participants:* adults with LD  *Gender:* 12 Canadian and 17 American men and 12 Canadian and 8 American women (n=49)  *Range of age:* 18 - 45  *Education:* 48 had a high school diploma, 1 a master degree  *Employment status*: all participants had previous or current work experience in their respective countries |
| Burns et al., 2013 [35] | A narrative, exploratory method [83] | To increase understanding of how tertiary teachers with dyslexia practice resilience strategies in work contexts and how those strategies might be associated with the practicalities of their profession | Narrative interviews lasting from an hour and a half to over two hours | *Participants:* teachers from Finland and all had been diagnosed in adulthood as having dyslexia  *Gender:* 3 men, 3 women (n=6)  *Range of age:* mid 30s to late 50s  *Education:* ranging from formal teacher’s qualifications, bachelor’s and master’s degrees to PhD  *Employment status:* all had a teaching position in tertiary education, varying from 3.5 to over 30 years |
| Raskind et al., 1997 [36] | An ethnographic or insider’s perspective with the researcher starting with a conscious attitude of almost complete ignorance [89] | To learn about assistive technology from the insiders, how technology can be used within the employment setting to compensate for LD. | Individual interviews with a 61-question interview protocol, designed to provide informants with a framework to ‘paint a picture’ of their lives and the ways they use technology within their work settings. | *Participants:* users of assistive technology with LD, on formal diagnostic evaluation.  *Gender:* 4 men, 1 woman (n=5)  *Range of age:* 32 - 60  *Education:* all had long histories of academic difficulties  *Employment status:* all were currently employed in ‘white -collar’ positions |
| Shessel and Reiff, 1999 [37] | The methodology of qualitative inquiry [90] | To examine the life experiences of adults with learning disabilities. The purposes of the study were to identify and further understand the positive and negative impacts and outcomes of learning disabilities in adulthood. Understanding these issues may lead to approaches that facilitate successful adjustment and adaptation to learning disabilities in adulthood. | Two in-depth interviews of approximately 1 – 1.5 hours each, based on the basic tenets of ethnographic interviewing [89] | *Participants: a*dults with dyslexia, recommended by the Learning Disabilities Association and from the Seneca College of Applied Arts and Technology in Toronto  *Gender:* 6 men, 8 women (n=14)  *Range of age:* 26 - 60  *Education*: full-scale IQ -scores from 86 to 117. Education levels between grade 11 to master’s degree  *Employment status:* occupations varied widely. Eight participants worked in ‘helping’ professions |
| Price and Gerber, 2001 [38] | Not mentioned | What impact has the ADA had on the American workplace, now that it is fully integrated into the culture of work? To explore this question, the findings of Gerber [91] at the very beginning of the ADA are compared to a study completed in 1998 and reported here. | Individual, on-site interviews with each employer, using a protocol with predetermined questions and open-ended probes to accurately reflect individual employer feedback [92;93] | *Participants: e*mployers / corporate representatives from Richmond, Virginia and the Delaware Valley in Philadelphia.  *Gender:* not mentioned (n=9)  *Range of age:* not mentioned.  *Education:* not mentioned.  *Employment status:* all were currently employed as supervisors, managers or human resource administrators in companies of various size. |
| Ferri et al., 2005 [39] | Narrative inquiry and critical discourse analysis [94;95] | In this investigation we examine how a specific group of individuals – teachers with LD – came to learn about their own disability and what sources of knowledge they relied on for this knowledge. The purpose is to untangle the voices, versions, visions of disability that have become embedded in the social discourse. | A series of three 60- to 90-minute in-depth interviews and a 2-hour final group meeting at the end of the data collection | *Participants:* teachers, all self-identified with LD  *Gender:* 3 men, 1 woman (n=4)  *Range of age:* 23 - 46  *Education:* not mentioned  *Employment status:* three were K-12 special education teachers and one student teacher. Years of experience: 8 - 16 |
| Macdonald, 2009 [40] | The study used primarily a qualitative biographical approach [96] | The study investigates the impact that disabling barriers have in education and employment for people with dyslexia, how issues of disabling barriers and social-class structures affect the lives of people with dyslexia. | Three individual interviews, in accordance with the biographical interpretative method of interviewing [96] | *Participants:* represent a continuum of individuals from different social backgrounds: 6 from the middle-class group and 7 from the working-class group. All participants had LD  *Gender:* 6 men, 7 women (n=13)  *Range of age:* 19 - 54.  *Education:* not mentioned  *Employment status:* 1 unemployed, 12 employed in various occupations |
| Duff et al., 2007 [41] | The study was conceived of as fundamentally exploratory in nature and very largely informed by the conceptual frameworks of the medical and social models of disability. | To consider attitudes of human resource managers towards the employment and employability of learning disabled people in United Kingdom accounting firms. | Semi-structured individual interviews, lasted between 30 minutes to one hour | *Participants:* HR-managers from UK accounting firms: five from Top-10 firms and four from firms ranked 11 to 50.  *Gender:* not mentioned (n=9)  *Range of age:* not mentioned  *Education:* not mentioned.  *Employment status*: 7 were employed as Human Resource Manager, 1 as Health and Safety Advisor/Occupational Health an 1 as partner. |
| Illingworth, 2005 [42] | A qualitative approach was taken comprising semi-structured interviews and interpretative data analysis using the process of coding [97] | To explore the effects of being dyslexic on the working lives of nurses and HCA’s and to identify what might be done to improve their working lives. | The semi-structured interviews included open-ended and direct questions, as well as dyslexia screening questions, lasted between 30 and 90 minutes and were conducted over 11 months. | *Participants:* nurses and healthcare assistants (HCA) who had been diagnosed with dyslexia or had dyslexic traits.  *Gender:* 3 men, 4 women (n=7)  *Range of age:* 23 to 43  *Education:* 3 nurses were studying for a first degree and 2 for a master’s degree.  *Employment status:* all 7 were working in a number of clinical areas in the trust. |
| Greenbaum et al., 1996 [43] | Not mentioned. | Not mentioned. | Telephone interviews with a protocol, consisting of 105 questions: 11 for demographic information, 22 on current and previous employment, 6 focused on social status, 65 concentrated on college experiences and 1 for additional information or comments. | *Participants:* previously undergraduate students at the University of Maryland between 1980 and 1992, with LD  *Gender:* 30 men, 19 women (n=49)  *Range of age:* the mean age at the time of the interviews was 26 years  *Education:* 44 had completed an undergraduate degree, 5 were still working on their undergraduate degree at institutions other than the University of Maryland  *Employment status:* 35 were employed, 7 unemployed and not in school, 7 were attending school full-time |
| Gilmour, 1998 [44] | Not mentioned. | The pilot of the Anger Management Programme had three main goals:  To develop a cognitive-behavioral based interaction group program for client use;  To collate an in-service training pack for facilitators;  To initiate collaborative primary care/social work team -working practices. | Clients were tested on standardized and non-standardized assessments which combined skill levels/subjective observations and formalized scoring. The facilitators’ perceptions of the Anger Management Programme were evaluated through questionnaires and semi-structured interviews. | *Participants:* adults with mild/moderate LD, a lack of interactional strategies/social use of language/communication difficulties, with invasive challenging behaviors.  *Gender:* 6 men, 4 women (n=10)  *Range of age:* 20 to 45  *Education:* not mentioned  *Employment status:* not mentioned |
