# Supplementary material for: Factors influencing work participation of adults with developmental dyslexia: a systematic review
Source: BMC Public Health. 2014 Jan 24;14:77. doi: 10.1186/1471-2458-14-77 (PMC3913008; doi:10.1186/1471-2458-14-77)
Supplement: Additional file 2 — Main characteristics of the quantitative studies [45-60,98-106]. [file 1471-2458-14-77-S2.doc]

Additional file 2: Main characteristics of the quantitative studies

| **Author(s)** | **Study design and setting** | **Aim of the study** | **Method for data collection** | **Statistical**  **analysis** | **Description and number of participants** | **Outcome measures** |
| --- | --- | --- | --- | --- | --- | --- |
| Mellard et al., 2007 [45] | Survey, cross-sectional  Participants were recruited from 12 of the 31 adult education programs in Kansas | To identify the relation of age, gender, education level, reading level, self-reported learning disability (LD) status and employment status to reading practices of individuals attending adult basic and secondary education programs | A structured interview that covered demographic characteristics, education, health, occupation, family histories, reading practices.  14 measures of literacy from the Woodcock Reading Mastery Test-revised [98] and the Comprehensive Adult Student Assessment System Reading [99] | Descriptive statistics,  Mantel-Haenzel chi-squared statistic, Pearson correlation and Mann-Whitney  Multivariate analysis | *Participants:* individuals attending adult basic and secondary education programs  *Gender:* 87 men,126 women (n=213)  *Range of age:* 16 – 59 (205), 60+: 8  *Education:* 187 participants had not completed high school, 26 completed 12^th^ grade or higher  *Employment status:* employed 167; unemployed 46 | Independent variables: age, education level before Adult Education, reading level, gender, employment status and self-reported LD-status  Dependant variables: reading practices scores, WRMT-R PC raw score, CASAS-Reading raw score |
| Madaus et al., 2003 [46] | Survey research, cross-sectional  Graduates from a large, competitive, public post-secondary institution in the Northeast between 1985 and 1999 | To determine levels of employment satisfaction of a sample of university graduates with LD and to determine what personal and work-based attributes contribute to these perceptions | A questionnaire that consisted of four sections: a demographic section, a section related to Job Satisfaction, to Self-Efficacy regarding employment and a section on the respondent’s university records | Confirmatory factor analysis was used to assess the psychometric properties of the instrument. Hierarchical regression analysis, descriptive statistics and Pearson’s correlation were employed | *Participants:* graduates with Learning Disabilities who voluntarily self-disclosed and submitted documentation that met the criteria for a specific learning disability.  *Gender:* 62 men, 27 women (n=89)  *Range of age:* 59 participants < 30, 30 > 30 years  *Education:* participants graduated from the College of Liberal Arts and Sciences (55%), the School of Education (11%), School of Family Studies (10%), College of Agriculture and Natural Resources (9%) and the School of Business Administration (9%)  *Employment status:* 78 participants were employed full-time. The areas of employment, most frequently reported, were business (30%), education (15%), health care (11%) and technology (11%). 12% was self-employed | The extent to which variance in employment satisfaction can be explained by a set of personal attributes, career and employment factors, learning disability and work experience factors and levels of employment self-efficacy |
| Dickinson & Verbeek, 2002 [47] | Cross-sectional  University of Arizona and the National Longitudinal Survey of Youth | To decompose wage differentials between adults with and without learning disabilities | Measurement of pay, tenure benefits (such as health, dental, life insurance, flexible hours, retirement benefits), multiple jobs and membership of a union | To estimate wage equations by Ordinary Least Squares (OLS) regression methods; standard linear regression methods | *Participants:* an original data set of the Strategic Alternative Learning Techniques (SALT) centre at the University of Arizona and a control sample of the 1994 National Longitudinal Survey of Youth (NLSY)  *Gender:* SALT 54 women, 43 men (n=97); NLSY 565 women, 565 men (n=1130)  *Range of age:* SALT 22 - 33; NLSY 28 - 33  *Education:* SALT 0,5 year of postgraduation education; NLSY 0,7 year  *Employment status:* SALT n=85; NLSY n=1079 | Wage differentials between college graduates with and without learning disabilities |
| Rojewski, 1999 [48] | Longitudinal study over 2 years  A sample of the National Education Longitudinal Study: 1988-1994; the third in an ongoing series of longitudinal studies for the educational, vocational and personal development of adolescents and young adults | To describe and compare the occupational and educational status of young adults with and without LD 2 years after high school completion, and to determine the predictive value of selected factors | The self-esteem scale (7 items) and the locus of control scale (6 items)  Standardized grade 12 achievement scores on reading, mathematics and science achievement (multiple choice items)  Educational aspirations were conceptualized at a 9-point interval-level construct.  Occupational aspirations were measured by choosing from 17 separate occupational categories. | Descriptive statistics; Log-Linear Analysis and Predictive Discriminant Analysis. | *Participants:* individuals who had participated in all four rounds of data collection in the National Education Longitudinal Study ‘88-‘94. They were all out of high school for a period of two years.  *Gender:* LD: 272 men. 161 women (n=433)  Non LD: 5253 men, 5484 women (n=10.737)  *Range of age:* 19 – 21 years  *Education:* all completed high school 2 years ago. Slightly less than one third of the men with LD was enrolled in some type of postsecondary education, compared to one half of men without LD. Within the women without LD more than half was enrolled, compared to a quarter of the women with LD  *Employment status:* two-third of the men with LD were in the workforce compared to less than one half of the men without LD. Within the women with LD the half in comparison to a third in the women without LD | Measures of personality: self-esteem and locus of control  Academic achievement: reading, mathematics and science achievement scores.  Educational aspirations  Occupational aspirations |
| Madaus et al., 2008 [49] | Survey, cross-sectional  Graduates from three postsecondary institutions nationwide | To determine levels of employment satisfaction and to explore the factors contributing to these perceptions using the prediction model previously employed by Madaus et al. (2003) | The final instrument consisted of four sections: 1. Demographic items; 2. Items related to the Americans with Disabilities Act; 3. Job satisfaction; 4. Employment self-efficacy | Descriptive statistics; hierarchical regression analysis [100] | *Participants:* graduates of three post-secondary institutions with a specific learning disability  *Gender:* 255 men, 245 women (n=500)  *Range of age:* mean: 31,3; SD: 6,0  *Education:* all participants were graduates at the included institutions or were still in pursuit of an additional degree  *Employment status:* the average length of time in a position was 3,5 years (SD = 3,0) | Participant’s perceptions of employment satisfaction and the factors contributing to these perceptions: employment self-efficacy, use of self-regulatory strategies, employment disclosure and demographic characteristics |
| Curtis Breslin & Pole, 2009 [50] | Data from cycle 2.1 of the Canadian Community Health Survey (CCHS), a large population based survey conducted in 2003  Canadian Community Health Survey (CCHS), conducted between January and September 2003 | To examine the association between the mental health conditions of young people with learning disabilities and ADHD, types of jobs held and the occupational injury experiences of Canadians aged 15 to 24 years | A questionnaire to assess all the variables mentioned | A logistic regression model to assess the relationship between learning disabilities, ADD/ADHD, school status, demographic characteristics, work variables and work-related injuries | *Participants:* CCHS respondents who reported having been employed in the preceding 12 months and who had been diagnosed with dyslexia or another type of learning disability, ADD or ADHD  *Gender:* 7382 men, 6997 women (n=14.379)  *Range of age:* 15 - 24  *Education:* not completed high school but currently enrolled in school (3331); Completed high school but not currently enrolled in school (4785); Completed high school and currently enrolled in school (5091); Not completed high school and not currently enrolled in school (1052)  *Employment status:* manual (5907), non-manual (4233) mixed (4116) | Diagnosis, school status, working hours per week, job type, age, gender, province or residence  Injuries identified as taking place during work and serious enough to limit the normal work activities |
| Witte et al., 1998 [51] | Survey, cross-sectional  All participants received their undergraduate degrees from Miami University, a competitive liberal arts institution, between 1987 and 1994 | To provide a more detailed examination of the perceived “fit”, as postulated in the Theory of Work Adjustment, of this working population with their work environment and their job satisfaction profiles | The survey form consisted of four sections: demographic information, employment, Office of Learning Assistance services, Job Satisfaction Scales (Work, Supervision, Colleagues, Promotion, Pay) | One-way ANOVA between groups and chi-square | *Participants:* undergraduate students who self-reported their learning disability (n=55) and controls from alumni records who matched with the LD students according to gender, major, degree, and graduation year (n=55)  *Gender:* LD-group 35 men and 20 women  *Mean age:* LD-group 25.7 (sd 7.4) and the controls 25.7 (sd 6.8)  *Education:* university students  *Employment status:* of the LD-group 48 persons were employed full-time, 5 part-time (in a great range of job categories), 1 unemployed and 1 was attending medical school. | Current employment and relative job satisfaction, examined according to several core dimensions: colleague and work relationships, actual work conditions, supervision of work, remuneration, job promotion and overall collective satisfaction |
| Vogel & Holt, 2003 [52] | A comparative study of six English-speaking populations who participated in the first administration of the International Adult Literacy Survey (IALS); cross-sectional survey; comparative cross-cultural research  The IALS-data were gathered in 1996. Further specifications of the setting are not mentioned | To explore the identified differences in light of the increase in recognition of the co-occurring disability of ADHD, in the predominant reading methodology in different countries and economic support available for education; to make recommendations to improve literacy proficiency and long-term outcomes for those with LD in all nations | Questions from the IALS on literacy levels, education and employment | Descriptive statistics were used; no inferential tests because of the lack of power and limited generalizability due to the small proportions of persons with SRLD. Population weights were used, normalized to the original sample sizes in each country. Percentages are reported for those with SRLD and non-SRLD. | *Participants:* participants of the IALS in 1996 in six English-speaking countries: English-speaking Canada, Great-Britain, Republic of Ireland, New Zealand, Northern Ireland and the United States (n=17.027; 805 self-reported a LD = SRLD and 16.222 NSRLD)  *Gender:* SRLD: 469 men, 336 women; NSRLD: 7886 men, 8335 women  *Range of age:* 16 - 65  *Education:* mean number of years of schooling for SRLD = 11.01 and for Non-SRLD = 12.29. High level of educational attainment (= high school graduation and beyond) for SRLD = 29.31% and Non-SRLD = 54.36%  *Employment status:* unemployed SRLD: 21.47% and Non-SRLD 9.48%. Employees in ‘white collar’-jobs: SRLD 32.19% and Non-SRLD 50.16%. Employees in ‘blue collar’-jobs: SRLD 67.81% and Non-SRLD 49.67% | Prevalence of SRLD, percentage by age, gender distribution, literacy proficiency, educational attainment, employment and income. |
| Taylor & Walter, 2003 [53] | Survey, cross-sectional  All participants came from Central Southern England and had a similar socio-economic status, although social class was not formally assessed | To investigate the types of differences in career choice between adults with and without symptoms of dyslexia, the occupations of adults with and without symptoms of DD were examined | Dyslexia rating with the Adult Dyslexia Organization 20-question checklist [101];  Occupation choices by coding them in the Standard Occupational Codes, developed by the United States Bureau of Labor Statistics in 2002 | Test for normality: Kolmogorov-Smirnov test.  Mann-Whitney U test to compare the mean scores between the groups and Fisher’s exact test to compare sex distribution between the dyslexic and control group.  Chi-square test for the distribution of occupational category between groups.  Odds-ratio for the relative proportions of dyslexics and controls in each category. | *Participants:* adult participants who were parents of children with dyslexia, underwent a screening with the ADO 20-question checklist. A score ≥ 9 is indicative of dyslexic type problems  *Gender:* persons with dyslexia: 91 men, 87 women. Controls: 83 men, 104 women (n=365)  *Mean age:* persons with dyslexia: 46.9, sd 6.98; Controls: 45.3, sd 5.72  *Education:* not mentioned  *Employment status:* 24 participants didn’t give an occupation, 28 defined their occupation in terms of parental role, the other 313 defined their profession | Occupation choices |
| Millward et al., 2005 [54] | A small exploratory study  The relationship between dyslexia traits and nurse performance on a laboratory task | To investigate the relationship between known dyslexia traits and performance on one particular ‘dyslexia sensitive’ task designed to simulate one of the key cognitive skills involved in drug administration | Learning Styles Questionnaire [102]  Accuracy Tasks [102]. A paired associated learning task  A self-report 30 item questionnaire  An additional questionnaire for demographic information | Descriptive statistics  Bivariate correlations  Regression analysis | *Participants:* 40 student nurses from a variety of stages in training and 6 qualified nurses  *Gender:* 6 men, 40 women (n=46)  *Range of age:* 18 – 51 with an average of 33  *Education:* not mentioned except the nursing education  *Employment status:* student nurses in training or employed as nurse | Presence of dyslexia traits  Performance on a cognitive task (i.e. matching patient names to drug names and vice versa)  Perceived performance control |
| Ingesson, 2007 [55] | Cross-sectional  A dyslexia clinic in Sweden | To investigate how a group of Swedish teenagers and young adults describe their youth, with special focus on the psychosocial experience of growing up with dyslexia | Semi-structured interview in which some questions could be answered by choosing an alternative which was described verbally and assigned a numerical value | Two-tailed non-parametric Spearman’s correlations and Student t-tests | *Participants:* Swedish teenagers and young adults  *Gender:*48 men, 27 women (n=75)  *Range of age:* 14 - 25  *Age at diagnosis:* mean age of 12 years  *Education:* 35% had secondary school, 30% had passed upper secondary school and 35% had a college degree  *Employment status:* temporary n=7; permanent n=15; unemployed n=4; other participants were still in education | Subject’s own experiences of self-esteem and peer-relations in school, the choice of education and subsequent occupation. |
| Morris & Turnbull, 2007 [56] | A survey-based exploration  Registered nurses with a confirmed dyslexia diagnosis, working in any discipline of nursing, within the UK | To explore the effects of dyslexia on the practice and career progression of UK registered nurses | A 12-item questionnaire requesting quantitative bio-demographical information and qualitative responses about the participant’s clinical experiences of dyslexia | Quantitative data were subjected to descriptive statistical analysis.  Content analysis provided the framework for analysing qualitative data | *Participants:* registered nurses with a confirmed dyslexia diagnosis (n=116)  *Gender:* not mentioned  *Range of age:* not mentioned  *Education:* D grade was the minimum level for registered nurses, rising to I grade, reflecting high levels of responsibility and autonomy  *Employment status:* 66 worked almost exclusively in community-based settings, 47 in acute care/hospital settings, 3 were employed in both community and acute care settings | Bio-demographical information  Participant’s clinical experiences of dyslexia |
| Madaus et al., 2002 [57] | Survey, cross-sectional  All participants attended and graduated from a competitive public institution in the Northeast between 1985 and 1999 | To examine the critical issue of self-disclosure and accommodation use in the workplace by postsecondary graduates with LD | A questionnaire that consisted of four sections: a demographic section, a section related to Job Satisfaction, to Self-Efficacy regarding employment and a section on the respondent’s university records | Percentages and clustering of qualitative responses | *Participants:* adults with Learning Disabilities who voluntarily self-disclosed and submitted documentation that met the criteria for a specific learning disability. All of them had participated in a formal academic support program for students with LD  *Gender:* 90 men, 42 women (n=132)  *Range of age:* 21 - 36  *Education:* participants graduated from the College of Liberal Arts and Sciences (55%), the School of Education (11%), School of Family Studies (10%), College of Agriculture and Natural Resources (9%) and the School of Business Administration (9%)  *Employment status:* 113 participants were employed full-time, 8 part-time and 11 were unemployed. The areas of employment most frequently reported were business (30,3%), education (14,6%) and health care and technology (11,2%). 12% was self-employed | Reasons for disclosure or non-disclosure and accommodation use in the workplace |
| Schulte-Körne et al., 2003 [58] | Measurement after a long term period of about 20 year of writing achievements and the social and mental developments  Oberurff is one of the 30 dyslexia-centres of the christian Jugenddorf-werkes in Germany | To assess a group of former students of a boarding school for dyslexic children (Chrisophorus School Oberurff) | Mannheim Writing Test [103],  Culture Fair Intelligence test (CFT 20) [104],  Symptom-checklist von Derogatis, SCL-90-R [105],  Magnitude-Prestige-Scale [106] | Descriptive statistics One-sample t-test  Signed-Rank-Test  Correlations | *Participants:* participants were recruted from the former students of the boarding school of the Christophorus School in Oberurff (Nord-Hessen). This research took place in 1998 among all students who graduated between 1980 and 1988  *Gender:* 27 men, 2 women (n=29)  *Range of age:* mean 31,2 ± 2,7  *Education:* Abitur/Fachabitur 41%  Secondary school 56%  Primary school 3%  *Employment status:* the occupations of the participants and of their parents were registered | The current writing achievement  Intelligence  Subjectively experienced mental and physical impediments  The occupations of the participants and of their parents |
| Chapman et al., 2003 [59] | Cross-sectional; a secondary analysis of the New-Zealand data from the International Adult Literacy Survey (IALS)  The IALS-data were gathered in 1996. Further specifications of the setting are not mentioned | To investigate the relation between the experience of specific reading learning disability and literacy proficiency, gender and age distribution, ethnic background, educational attainment, and economic outcomes | Questions from the IALS on literacy levels, education and employment | Weighted population percentage estimates within the Specific Reading Learning Disability (SRLD) – group, the non-SRLD-group and the total population | *Participants:* a subsample from the New-Zealand IALS-sample. They self-reported a learning disability  *Gender:* 123 men, 82 women (n=205)  *Range of age:* 16 - 65  *Education:* 180 participants completed high school, 24 completed post-high school qualifications  *Employment status:* 33 participants were unemployed, 172 employed in a great range of occupations | Incidence of specific reading/ learning disability (SRLD)  Relation of SRLD to literacy proficiency, gender, age, ethnic background, educational attainment, economic outcomes and methods of literacy instruction |
| Magajna et al., 2003 [60] | Cross-sectional; a secondary analysis of the Slovenian data from the International Adult Literacy Survey (IALS)  The IALS-data were gathered in 1996. | To interpret the IALS findings for Slovenia in terms of prevalence, age and gender, literacy skills, educational attainment, employment and everyday literacy activities of adults with SRLD | The literacy measures for prose, document and quantitative literacy; the IALS questionnaire | Descriptive statistics; weighted population percentage estimates within the Specific Reading Learning Disability (SRLD) – group and the non-SRLD-group | *Participants:* a subsample of the Slovenian IALS-sample; all had reported experiencing learning disabilities  *Gender:* 37 men, 42 women (n=79)  *Range of age:* 16 - 65  *Education:* participants have on average only 8 years of schooling  *Employment status:* all participants were employed | Interpretation of the Slovenian IALS data in terms of prevalence, age and gender, literacy skills, educational attainment, employment and everyday literacy activities |
